# Thoracic Aortic Aneurysm Development in Patients with Bicuspid Aortic Valve: What Is the Role of Endothelial Cells?

**DOI:** 10.3389/fphys.2017.00938

**Published:** 2017-11-30

**Authors:** Vera van de Pol, Kondababu Kurakula, Marco C. DeRuiter, Marie-José Goumans

**Affiliations:** ^1^Department of Molecular Cell Biology, Leiden University Medical Center, Leiden, Netherlands; ^2^Department of Anatomy and Embryology, Leiden University Medical Center, Leiden, Netherlands

**Keywords:** bicuspid aortic valve, thoracic aortic aneurysm, endothelial cells, endothelial-to-mesenchymal transformation, transforming growth factor beta, angiotensin II, nitric oxide, notch1

## Abstract

Bicuspid aortic valve (BAV) is the most common type of congenital cardiac malformation. Patients with a BAV have a predisposition for the development of thoracic aortic aneurysm (TAA). This pathological aortic dilation may result in aortic rupture, which is fatal in most cases. The abnormal aortic morphology of TAAs results from a complex series of events that alter the cellular structure and extracellular matrix (ECM) composition of the aortic wall. Because the major degeneration is located in the media of the aorta, most studies aim to unravel impaired smooth muscle cell (SMC) function in BAV TAA. However, recent studies suggest that endothelial cells play a key role in both the initiation and progression of TAAs by influencing the medial layer. Aortic endothelial cells are activated in BAV mediated TAAs and have a substantial influence on ECM composition and SMC phenotype, by secreting several key growth factors and matrix modulating enzymes. In recent years there have been significant advances in the genetic and molecular understanding of endothelial cells in BAV associated TAAs. In this review, the involvement of the endothelial cells in BAV TAA pathogenesis is discussed. Endothelial cell functioning in vessel homeostasis, flow response and signaling will be highlighted to give an overview of the importance and the under investigated potential of endothelial cells in BAV-associated TAA.

Bicuspid aortic valve (BAV) is the most common congenital cardiovascular malformation with a prevalence of 0.5–1.5% in the general population and a male predominance of about 3:1 (Roberts, 1970; Basso et al., 2004). In this anomaly, the aortic valve consists of 2 leaflets instead of the regular 3 leaflets. The BAV usually exhibits normal function at birth and during early life, however in adulthood BAV patients can develop several serious complications such as valvular stenosis and/or regurgitation, aortic dilation, and thoracic aortic aneurysms (TAA). Although TAAs occur both in tricuspid aortic valves (TAV) and BAV, it has been estimated that 50–70% of BAV patients develop aortic dilation and ~40% of BAV patients develop TAAs (Yuan et al., 2010; Saliba and Sia, 2015). Moreover, patients with a BAV have a 9-fold higher risk for aortic dissection compared to the general population (Lewin and Otto, 2005). To monitor dilation progression in BAV patients the aortic diameter is regularly measured using echocardiography. However, no treatment options are available to prevent dilation or impact on the remodeling aortic wall. Surgical intervention with the aim to prevent rupture is therefore currently the only therapy for TAAs.

## Thoracic aortic aneurysm

**Figure 1:**
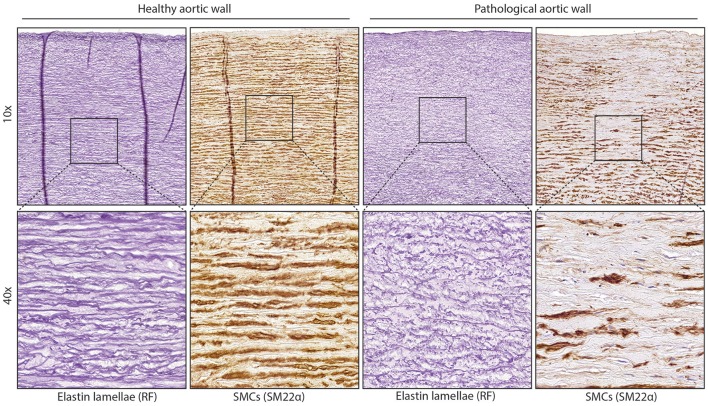
Structure of normal and diseased aortic wall. Images of aortic tissue showing elastic lamellae (stained with RF) or smooth muscle cells (SM22 staining) On the left is normal aortic tissue, the right image shows aortic tissue with fragmentation of the lamellae or loss of contractile SMCs.

While smooth muscle cells (SMCs) in the healthy media have a contractile phenotype, they are not terminally differentiated. This ensures the ability to regenerate the vessel wall after injury. This flexible change between cellular phenotypes is called “phenotypic switching,” with the contractile and synthetic SMCs on opposite sides of the spectrum. After phenotypic switching the synthetic SMCs can migrate towards a wounded area by secreting proteinases to break down the ECM. Synthetic SMCs also proliferate and produce ECM to repair the wall. When the vessel wall is repaired, synthetic SMCs will re-differentiate toward a contractile phenotype. TAA is characterized by phenotypic switching of contractile to synthetic SMCs and fragmentation of elastic lamellae (Figure 1). The BAV aorta is more prone to TAA development, possibly due to differences in vascular homeostasis. For example, it has been shown that non-dilated BAV aorta, like the dilated TAV aorta, has an increased collagen turnover (Wågsäter et al., 2013). Moreover, orientation, fiber thickness, and collagen crosslinking is altered in the dilated BAV aorta compared to the TAV aorta (Tsamis et al., 2016). Additionally, decreased expression levels of lamin A/C, α-smooth muscle actin (α-SMA), calponin, and smoothelin were not only found in dilated, but also in non-dilated BAV aorta (Grewal et al., 2014). Abdominal aortic aneurysms (AAA) share some common features with TAA, but differ in that atherosclerosis plays a major role in AAA, whereas medial degeneration is characteristic of TAA (Guo et al., 2006).

The mechanism initiating thoracic aortic dilation is thus far unknown, however, the two main hypotheses are that either an altered flow greatly impacts vessel wall homeostasis (flow hypothesis) or that an intrinsic cellular defect contributes to the formation of BAV as well as to the dilation of the aorta in these patients (genetic hypothesis; Girdauskas et al., 2011a). Several genes related to structural proteins have been found mutated in BAV patients, such as *ACTA2, MYH11*. Furthermore, in BAV patients multiple mutations have also been found in genes related to signaling proteins such as *NOTCH1* and genes related to the TGFβ signaling pathway (Girdauskas et al., 2011b; Tan et al., 2012; Andelfinger et al., 2016). In addition to isolated cases, BAV has also been demonstrated to occur within families (Huntington et al., 1997; Calloway et al., 2011). Interestingly, 32% of the first-degree relatives of BAV patients with a TAV also develop aortic root dilation, suggesting that the genetic predisposition for BAV and TAA overlap or may be identical in these families (Biner et al., 2009). However, a clear inheritance pattern remains to be found. TAAs are also observed in patients with other syndromes such as Marfan, Loeys–Dietz, and Ehler–Danlos, but contrastingly, BAV seldom occurs in these syndromes (El-Hamamsy and Yacoub, 2009; Ruddy et al., 2013). For an overview of genetic variation associated with BAV and the effect on endothelial functioning see Table 1.

**Table 1:** Consequences of genetics associated with BAV on cardiac malformations and endothelial cell functioning.

**Pathway**	**Mutation**	**Effect**	**Other cardiovascular malformations**	**BAV occurrence**	**Effect of mutation on endothelial function**
TGFb	*Gata5*^cre^*Alk2*^fl/fl b^ (Thomas et al., 2012)	ALK2 deletion in cushion mesenchyme	not/under developed non-coronary leaflet	78–83%	Constitutively active ALK2 induces EndoMT and is required for HDL induced EC survival and protection from calcification (Yao et al., 2008; Medici et al., 2010)
	*ENG*^a^ (Wooten et al., 2010)	Conservative peptide shift	HHT	Increased haplotype in BAV with an OR of 2.79	Flow and ligand induced EC migration is disrupted increased proliferation and responsiveness to TGFβ1 (Pece-Barbara et al., 2005; Jin et al., 2017)
	*TGFBR2*^a^ (Attias et al., 2009; Girdauskas et al., 2011b)	Missense/nonsense/splicing mutations	LDS, Marfan, TAA	7% of the cohort	Maintenance of vascular integrity (Allinson et al., 2012)
	*SMAD6^a^* (Tan et al., 2012)	Loss of function	AoS, AoC, and aortic calcification	3/436 patients, 0/829 controls	Increases SMAD6, inhibits TGFβ signaling (Topper et al., 1997)
	*Adamts5*^−/−^*Smad2*^+/−b^ (Dupuis et al., 2013)	Loss of function for Adamts5 and SMAD2	Myxomatous valves, BPV	75% Non-coronary with either left or right coronary cusp	Embryonic vascular instability, SMAD2 increases eNOS expression (Itoh et al., 2012)
Other	*Ift88*^fl/fl^*Nfatc*^Cre b^ (Toomer et al., 2017)	Endothelial specific loss of primary cillia	–	68% BAV right/non-coronary fusion	ECs without primary cilia undergo EndoMT upon shear stress (Egorova et al., 2011)
	*eNOS*^−/−b^ (Lee et al., 2000)	No functional eNOS	–	42% BAV right/non-coronary fusion	Decreased EndoMT (Förstermann and Münzel, 2006)
	*Gata5*^a^ /*Tie2*^cre^*Gata5*^fl/fl b^ (Laforest and Nemer, 2012; Bonachea et al., 2014; Shi et al., 2014)	Reduced Gata5 activity Gata5^a^/Gata5 deletion in ECs^b^	VSD, aortic stenosis^a^ / LV hypertrophy, AS^b^	autosomal dominant BAV inheritance^a^ / 25%^b^	Altered PKA and NO signaling (Messaoudi et al., 2015)
	*NOTCH1*^a^ (Garg et al., 2005)	Autosomal dominant mutant notch1	CAVD and other cardiac malformations	Autosomal dominant inheritance with complete penetrance	NOTCH1 increases calcification, oxidative stress and inflammation, when exposed to shear stress (Theodoris et al., 2015)
	*NKX2.5^a^* (Qu et al., 2014)	Loss of function	ASD, PFO, AS and conduction defects	One family with an autosomal dominant inheritance	–
	*ACTA2^a^* (Guo et al., 2007)	Missense mutation	Family with FTAAD	3/18 patients with TAAD and mutation	–
	*FBN1^a^* (Attias et al., 2009)	Diverse	Marfan, TAA	4% of the cohort	–

a *Found in human*.

b *Found in mice*.

*OR, Odds ratio; AoC, Aortic coarctation; AoS, Aortic valve stenosis; AS, Aortic stenosis; ASD, Atrial septal defect; BPV, Bicuspid pulmonary valve; CAVD, calcific aortic valve disease; HHT, Hereditary hemorrhagic telangiectasia; LDS, Loeys-Dietz syndrome; LV, Left ventricle; PFO, Patent foramen ovale*.

## Endothelial cells in vessel homeostasis

Due to the obvious medial degeneration in the aortic wall, research in the past decades has focussed on characterizing the organization and SMC phenotype of the aortic media during dilation and aneurysm (Wolinsky, 1970; Halloran et al., 1995; Ruddy et al., 2013). Therefore, despite their main regulatory function, endothelial cells have so far taken the back seat in research toward understanding and treating aortic dilation. However, there is growing evidence that endothelial cells play an important role in the development and progression of aortic dilation.

**Figure 2:**
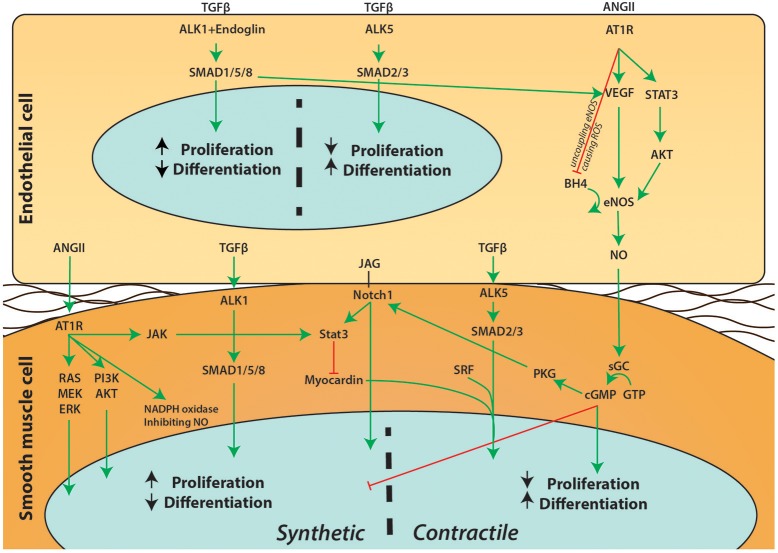
Schematic overview of signaling pathways between endothelial cells and SMCs. A simplified overview on the communication between endothelial cells and SMCs is depicted. Extensive crosstalk between pathways such as Notch1, ANGII, TGFβ, and NO can influence proliferation and differentiation of SMCs and affect the phenotypic switch of SMCs.

Endothelial cells line the lumen of the aorta which, together with some ECM and the internal elastic lamella, form the intima. As the layer between the blood (flow) and the main structural component of the aorta (the media) the function of endothelial cells is to communicate the signal between these two layers. Upon flow and stimuli such as inflammatory cytokines, signaling pathways like TGFβ, angiotensin, and nitric oxide (NO) allow endothelial cells to directly target the contraction status of SMCs or indirectly target the SMC contractile phenotype to influence vessel wall functioning (Figure 2). Primary cilia on the luminal surface of the endothelial cells enable mechanosensing and signaling (Egorova et al., 2012). Endothelial cells lacking cilia change toward a mesenchymal phenotype, a process called endothelial to mesenchymal transformation (EndoMT) in which endothelial specific genes such as VE-cadherin and PECAM1 are down-regulated, whereas mesenchymal genes such as αSMA and fibronectin are up-regulated (Egorova et al., 2011). Intriguingly, a recent study demonstrated that *Ift88*^*fl*−*fl*^ mice crossed with *Nfatc*^*Cre*^, thereby lacking a primary cilium specifically in endothelial cells, display a highly penetrant BAV (Toomer et al., 2017; Table 1).

## The influence of flow on endothelial functioning and vessel homeostasis

The flow pattern of blood from the heart into the aorta is altered by a BAV (Barker et al., 2012). This difference between TAV and BAV hemodynamics in the aorta can be beautifully demonstrated using 4D MRI. Compared to a TAV, BAV generate a high velocity “jet” propelling at an angle against the wall in the BAV aorta. This jet stream also causes an increase in peak shear stress on the endothelial cells (Barker et al., 2012). As mentioned above, aside from the genetic hypothesis, the altered flow is also hypothesized to cause the aortic dilation in BAV.

It has been long known that adjusting flow induces remodeling of the vessel wall. Already, more than 30 years ago it was published that by decreasing blood flow in the carotid artery of rabbits by 70%, the lumen size of the vessel was decreased by 21% to compensate for the decreased blood flow (Langille and O'Donnell, 1986). Vascular remodeling is induced by increased shear stress on endothelial cells to restore original shear forces on the wall (Baeyens et al., 2016a). That flow greatly impacts endothelial functioning is also portrayed by the localization of fatty streaks and atherosclerosis at branch points and curves of arteries (Baeyens et al., 2016a). The turbulent flow at these locations causes dysfunctional endothelium: endothelial cells undergo apoptosis or exhibit increased proliferation. Moreover, permeability is increased, allowing LDL penetration into the intima. In addition, inflammatory cell adhesion and infiltration is increased. Laminar flow induces the opposing quiescent endothelial phenotype characterized by a low turnover, alignment in the direction of the flow, decreased expression of inflammatory adhesion molecules like I-CAM and a low permeability caused by increased cell-cell adhesion molecules such as N-CAM and E-cadherin (Chistiakov et al., 2017). Experiments using co-culture of endothelial cells and SMCs revealed that flow on endothelial cells can also impact the phenotype of the underlying SMCs. Laminar shear stress on endothelial cells induces a contractile phenotype in synthetic SMCs, shown with both co-culture experiments of endothelial cells under flow with SMCs, as by adding conditioned medium from flow exposed endothelial cells to SMCs (Tsai et al., 2009; Zhou et al., 2013). Upon laminar flow, endothelial cells signal toward SMCs using, for example, microRNA (miR)-126, prostacyclin, TGFβ3, and NO (Noris et al., 1995; Tsai et al., 2009; Walshe et al., 2013; Zhou et al., 2013) MiR-126 in endothelial microparticles (EMPs) decreases SMC proliferation and neointima formation (Jansen et al., 2017). Interestingly, EMP secretion is elevated in BAV associated TAA (Alegret et al., 2016). It is believed that EMPs are formed when endothelial cells are trying to avoid undergoing apoptosis, possibly explaining the association of elevated levels of EMPs with vascular diseases such as diabetes, congestive heart failure and acute coronary syndrome (Rössig et al., 2000; Bernal-Mizrachi et al., 2003; Tramontano et al., 2010).

MiR-126 is only one means by which endothelial cells can impact on the vascular homeostasis. The main signaling pathways involved in BAV TAA and endothelial cells will be discussed in the next paragraphs.

## Angiotensin II signaling in TAA

One of the major signaling pathways disturbed in aortic dilation is the Renin-Angiotensin-Aldosterone-System (RAAS), which is important for maintaining blood pressure. By constriction/relaxation of blood vessels and adjusting water retention of the kidneys, the blood pressure is regulated. The juxtaglomerular cells in the kidney and baroreceptors in vessel wall can sense arterial blood pressure. Upon a drop in pressure, renin is released by the juxtaglomerular cells and renin then converts angiotensinogen into angiotensin I (ANGI), which in turn is converted by angiotensin converting enzyme (ACE) into angiotensin II (ANGII). Amongst others, ANGII can cause contraction of the SMCs to increase blood pressure. This contraction is caused by the binding of ANGII to the angiotensin II type 1 receptor (AT1) on the SMCs, resulting in activation of the Ca^2+^/calmodulin pathway, activating the myosin ligh chain kinase followed by rapid phosphorylation of MLC, causing contraction of SMCs. In addition, ANGII stimulates the cortex of the adrenal gland to secrete aldosterone, which increases water resorption in the kidney.

Aside from this direct vasoconstrictive effect, prolonged RAAS activation has diverse pathological effects. Aldosterone has been shown to cause endothelial dysregulation as well as a synthetic phenotype in SMCs (Hashikabe et al., 2006). Chronic infusion of ANGII in *ApoE*^−/−^ mice demonstrated to cause progressive TAAs and AAAs (Daugherty et al., 2000, 2010). The administration of ANGII in these mice decreased αSMA and calponin expression in the mouse aortas (Leibovitz et al., 2009; Chou et al., 2015). Moreover, ACE2 expression was increased in mouse aortas after ANGII infusion as well as in dilated aortas of BAV patients (Patel et al., 2014). ACE insertion/deletion polymorphisms were also identified as risk factor for the development of TAA in BAV patients (Foffa et al., 2012). Furthermore, a correlation was found between chronic elevated levels of ANGII and endothelial cell dysfunction in patients with hyperaldosteronism, underlining the importance of the RAAS system and endothelial functioning (Matsumoto et al., 2015).

A seminal study performed by Rateri and colleagues, displayed the importance of endothelial cell functioning in the ANGII aneurysm model (Rateri et al., 2011). Interestingly, mice with specific deletion of *AT1* in SMCs or monocytes still developed aortic aneurysms following a chronic ANGII infusion, while mice with an endothelial specific knock-out of *AT1* did not exhibit dilation of the thoracic aorta. This study indicates that the primary target cell for ANGII in this model is the endothelial cell, which in turn influences the SMCs, causing the aortic structure to break down. How exactly this ANGII-endothelial cell signaling affects the SMC phenotype remains a crucial and intriguing question to be investigated. The same group 1 year later showed that AAA are not inhibited in the endothelial cell specific *AT1* knock-out, elegantly demonstrating that indeed there is a difference in pathogenesis between TAA and AAA (Rateri et al., 2012). This difference might be explained by a more prominent role for the adventitia than the intima in AAA development, or the developmentally different origin of SMCs in different parts of the aorta (Police et al., 2009; Tieu et al., 2009; Tanaka et al., 2015; Sawada et al., 2017).

Aside from studies to understand the pathogenesis of TAA, ANGII treatment to model aortic aneurysm in mice is also used in the search of new treatment options. A recent study reported that treating ANGII infused mice with a combination therapy of Rosuvastatin and Bexarotene (retinoid X receptor-a ligand) inhibited aneurysm formation (Escudero et al., 2015). Moreover, they showed that this combination therapy affected endothelial cell proliferation, migration and signaling. In addition, upon ANGII treatment the VEGF secretion by endothelial cells *in vitro* was decreased (Escudero et al., 2015). SMCs from BAV patients exhibited an increased AT1R expression *in vitro*, which was reduced to the levels of control SMCs after treatment with losartan (Nataatmadja et al., 2013). Interestingly, antagonizing TGFβ by blocking the AT1 receptor using Losartan in a Marfan disease model mouse (*FBN1* mutation) demonstrated promising results for preventing and even reversing aortic dilation (Habashi et al., 2006). Furthermore, several clinical studies in Marfan patients reveal similar exciting results. However, a meta-analysis of clinical studies toward Losartan in Marfan patients did not show a reduction of aortic dilation in Losartan treated patients (Gao et al., 2016). Losartan treatment in BAV patients has not been investigated yet. A clinical study was initiated, but recently terminated due to low enrolment^1^. Therefore, the effect of Losartan on BAV TAA still needs to be determined.

## Notch1 signaling in TAA

Notch signaling plays an important role in cardiovascular development (Niessen and Karsan, 2008). In contrast to many signaling pathways, Notch signaling is cell-cell contact dependent. There are 4 Notch homologs of which Notch1 is the best known. Binding of Notch1 ligands Jagged1, Jagged2, and/or Delta expressed in one cell induces cleavage of the receptor and nuclear translocation of the intracellular domain in the other cell causing transcription of, amongst others, the HES/HEY gene family, key regulators in EndoMT (Noseda et al., 2004). Notch1 signaling induces EndoMT in endothelial cells and promotes a contractile phenotype in SMCs (Tang et al., 2010). Moreover, Notch1 signaling is required for angiogenesis (Krebs et al., 2000).

Notch1 signaling was displayed to be crucial for normal development of the aortic valve and outflow tract amongst others, as determined in *Notch1*^−/−^ mice (High et al., 2009). Specifically in the neural crest cells, Notch1 signaling is important. It was found that disruption of endothelial Jagged1 signaling to Notch1 on neural crest cells, inhibits SMC differentiation (High et al., 2008). The Notch1 signaling pathway, as well as the TGFβ signaling pathway, is involved in EndoMT occurring in the outflow tract cushions, where endothelial cells assume a mesenchymal cell phenotype to populate the developing cardiac valves (Niessen et al., 2008). Thereby EndoMT is a crucial part of aortic valve development. Previous studies hypothesized that EndoMT may also play a role in the pathogenesis of BAV. Additionally, genes involved in this process such as *NOTCH1, TGFBR2*, and *SMAD6*, have been found to cause BAV in mouse models, as well as being linked to BAV in human studies (Garg et al., 2005; Girdauskas et al., 2011b; Tan et al., 2012; Andelfinger et al., 2016; Gillis et al., 2017; Koenig et al., 2017). Mice with *Notch1* missense alleles have been characterized with multiple outflow tract and EndoMT defects (Koenig et al., 2015). Recently, it was demonstrated that specifically endothelial Notch1 signaling is required for normal outflow tract and valve development (Koenig et al., 2016). Moreover, a *Notch1* mutation was found in a family with BAV, underscoring Notch1 as an important signaling pathway in BAV (Garg et al., 2005). These mutations have been associated with an increased risk of calcific aortic valve disease (CAVD), explained by the normally repressive function of Notch1 on calcification in valvular cells (Garg et al., 2005; Nigam and Srivastava, 2009; Kent et al., 2013). Additionally, one study reported severely calcified valves in BAV patients with Cornelia de Lange syndrome, a disease caused by dysfunctional Notch1 signaling (Oudit et al., 2006).

Aside from the role of Notch1 signaling in valve formation, proper Notch1 signaling is also important for the homeostasis of the aorta, as illustrated by several studies. The non-dilated aorta of BAV patients showed increased Notch1 signaling and EndoMT marker expression based on proteomic analysis (Maleki et al., 2016). Furthermore, a study using endothelial cells isolated from BAV-TAA aorta demonstrated decreased *Notch1, Notch4*, and *Dll4* mRNA levels compared to TAV non-aneurysmal tissue (Kostina et al., 2016). Moreover, upon TGFβ stimulation, there was a defective Notch1 dependent EndoMT response. Endothelial marker proteins such as VWF and PECAM, were unchanged between BAV and TAV endothelial cells. However, EndoMT markers HES1 and SLUG were significantly less upregulated in BAV endothelial cells compared to TAV endothelial cells. In addition, *JAG1* expression is normally upregulated upon Notch1 signaling and acts as a positive feedback-loop. This upregulation of Jagged1 was decreased in BAV endothelial cells, explaining at least part of the dysfunctional Notch1 signaling in BAV patients with TAA (Kostina et al., 2016).

Interestingly, Notch1 plasma levels in combination with TNFα-converting enzyme were shown to correlate highly with the presence of AAA (Wang et al., 2015). Furthermore, studies demonstrated that *NOTCH1* haploinsufficiency or Notch1 inhibition can prevent or reduce the formation of AAA in ANGII infused mice (Hans et al., 2012; Cheng et al., 2014). However, the similarity in Notch signaling between AAA and TAA is debatable, as it has been displayed that in descending TAA tissue, in contrast to the ascending TAA, the SMCs exhibit a decreased Notch1 signaling, emphasizing the importance of the local environment in the aortic aneurysm formation (Zou et al., 2012).

## eNOS signaling in TAA

Nitric oxide (NO) is produced when NO synthase (NOS) converts arginine into citrulline, releasing NO in the process. NOS was originally discovered in neurons (nNOS/NOS1), after which inducible NOS (iNOS/NOS2) and endothelial NOS (eNOS/NOS3) were found. eNOS phosphorylation increases NO production and is induced by factors such as shear stress, acetylcholine and histamine. NO has a very short half-life of a few seconds, making it a local and timely signal transducer. Endothelial secreted NO diffuses into the SMC where it relaxes the cell by increasing the calcium uptake into the sarcoplasmic reticulum: NO stimulates the sarco/endoplasmic reticulum ATPase (SERCA), and thereby decreases cytoplasmic Ca^+^ levels (Van Hove et al., 2009). Additionally, NO has also been revealed to regulate gene transcription by reacting with NO sensitive transcription factors (Bogdan, 2001). Finally NO has been shown to impact the SMC inflammatory status, however more research is required to fully understand the effect of NO on SMC phenotype (Shin et al., 1996). Uncoupled eNOS causes free oxygen radicals to be formed, which damages proteins and DNA.

Multiple studies have identified an important role for dysregulated endothelial NO signaling in aneurysm development. For example, it has been demonstrated that the oxidative stress is increased in the media of the aortas of BAV patients compared to TAV aortas (Billaud et al., 2017). Interestingly, a mouse model with uncoupled eNOS (HPH-1 mice) rapidly developed AAA and aortic rupture upon ANGII infusion, whereas wild-type (WT) mice did not display this phenotype (Gao et al., 2012). Re-coupling of eNOS by infusion of folic acid, inhibited AAA formation (Gao et al., 2012). A study investigating the effect of iNOS deletion in an elastase infusion mouse model of experimentally induced AAA did not demonstrate any substantial exacerbation of the aneurysm phenotype, indicating the importance of endothelial NO in aneurysm formation (Lee et al., 2001). Intriguingly, a later study identified plasma and tissue levels of the eNOS co-factor tetrahydrobiopterin, necessary for coupling of eNOS, correlate with aneurysm development in *ApoE*^−/−^ mice and HPH-1 mice (Siu and Cai, 2014). In line with these studies, it was shown that endothelial specific expression of reactive oxygen species, by an endothelial specific overexpression of NOX2, can cause aortic dissection in WT mice upon ANGII infusion (Fan et al., 2014). Moreover, eNOS knockout mice develop BAV, underlining the importance of endothelial dysfunction in the formation of BAV and the related TAA (Lee et al., 2000).

In patients with a TAV and TAA, profiling of the aortic tissue revealed that eNOS phosphorylation was increased via a miR-21 dependent mechanism (Licholai et al., 2016). MiR-21 is specifically upregulated by shear stress and causes PTEN mRNA degradation, allowing an increase in eNOS phosphorylation (Weber et al., 2010). Furthermore, BAV TAA patient aortic samples displayed increased eNOS expression and activation compared to TAV TAA controls (Kotlarczyk et al., 2016). These studies indicate an increased eNOS activity in TAA formation in BAV patients. Contrastingly, decreased eNOS expression has been found in 72.7% aortic samples of BAV patients (*N* = 22; Kim et al., 2016). In addition, a negative correlation between eNOS expression levels and aortic dilation in BAV patients was reported (Aicher et al., 2007).

In conclusion, multiple studies have investigated eNOS in the BAV aorta, with contrasting outcomes (Aicher et al., 2007; Mohamed et al., 2012; Kim et al., 2016; Kotlarczyk et al., 2016). These discrepancies may be caused by differences between patient populations, location of the aortic sample used, stage of aortic aneurysm formation and the use of different control samples for comparison. Nonetheless, all these studies indicate that normal levels of coupled eNOS are necessary to maintain a healthy aortic wall.

## TGFβ signaling in TAA

TGFβ signaling is mediated by binding of the ligand TGFβ to the TGFβ type 2 receptor, which recruits and phosphorylates a TGFβ type 1 receptor. While there is only one type 2 receptor, TGFβ can signal via two TGFβ type 1 receptors, Activin-like kinase (ALK)1 and ALK5. Upon ligand binding, ALK5 can phosphorylate SMAD2 or SMAD3 and ALK1 can phosphorylate SMAD1, SMAD5 or SMAD8. The phosphorylated SMADs translocate into the nucleus with SMAD4 to induce the canonical signaling pathway. TGFβ can also signal via non-canonical pathways by activating PI3K/AKT, MAPK, or NF-kB. Via the canonical and non-canonical pathways, TGFβ influences cell cycle arrest, apoptosis, inflammation, proliferation, and more.

In endothelial cells, TGFβ signaling can either inhibit or stimulate the cell growth and function depending on the context (Goumans and Ten Dijke, 2017). TGFβ signaling via ALK1 induces proliferation and migration, whereas ALK5 signaling promotes plasminogen activator inhibitor 1 (PAI1) expression, decreasing breakdown of ECM which is necessary for maturation of the vessel wall (Goumans et al., 2002; Watabe et al., 2003). The two opposing effects of TGFβ signaling enable the initial growth of vessels followed by stabilization of the ECM and attraction of SMCs. Moreover, endothelial TGFβ signaling in concert with platelet derived growth factor-BB is crucial for attracting and differentiating pre-SMCs during vasculogenesis (Hirschi et al., 1998). Because of these crucial functions of TGFβ during embryonic development, loss of TGFβ signaling in the vascular system, either total knockout, SMC or endothelial cell specific deletion is embryonically lethal (Goumans and Ten Dijke, 2017). In SMCs TGFβ induces a contractile phenotype, and dysregulation of TGFβ therefore can have a major impact on SMC phenotype (Guo and Chen, 2012). The importance of endothelial TGFβ signaling on SMC differentiation is illustrated by co-culture of endothelial cells and SMCs. Cultured alone, the SMCs have a synthetic phenotype, but when co-cultured with endothelial cells, they differentiate into contractile SMCs via the PI3K/AKT signaling pathway (Brown et al., 2005).

The TGFβ Type III receptor endoglin (*ENG*) is highly expressed by endothelial cells and plays a role in the ALK1 and ALK5 signaling balance (Goumans et al., 2003). In fact, without endoglin, endothelial cells stop proliferating as a result of decreased ALK1 signaling (Lebrin et al., 2004). In addition, knock-out of *ENG* in mice causes embryonic lethality due to impaired angiogenesis, whereas vasculogenesis remains intact (Li et al., 1999; Arthur et al., 2000). This exemplifies the pivotal role for TGFβ signaling in endothelial cells for proper angiogenesis. As mentioned above, TGFβ signaling, like Notch1 signaling, is important for the process of EndoMT necessary for the developing cardiac valves. Chimera research using *ENG*^−/−^ mice embryonic stem cells, added to WT mice morulae highlighted the indispensable role of endoglin for EndoMT in the developing cardiac valves (Nomura-Kitabayashi et al., 2009). These chimeric mice showed contribution of the *ENG*^−/−^ cells to the endothelium. However, no *ENG*^−/−^ cells participated in populating the atrio-ventricular (AV) mesenchyme of the developing AV cushions. Intriguingly, a single-nucleotide polymorphism in *ENG* was found in BAV patients, indicating that in BAV patients endothelial TGFβ signaling might be altered, potentially promoting a phenotypic switch in the underlying SMCs (Wooten et al., 2010).

Many studies using *in vitro, ex vivo* and histological methods, also indicate a role for TGFβ signaling in TAA formation in BAV. Unstimulated, cultured BAV and TAV SMCs did not demonstrate any difference in gene expression in basal conditions, however after TGFβ stimulation, 217 genes were found differentially expressed between BAV and TAV SMCs demonstrating a difference in TGFβ signaling (Paloschi et al., 2015). Moreover, induced pluripotent stem cells (iPSCs) derived from BAV patients with a dilated aorta exhibited decreased TGFβ signaling compared with iPSCs from TAV controls without aortic dilation (Jiao et al., 2016). Conversely, a hypothesis-free analysis of the secretome of BAV TAA indicated a highly activated TGFβ signaling pathway in the aortic wall of BAV patients when compared to the secretome of TAV aneurysmal aortic tissue (Rocchiccioli et al., 2017). This study showed, using mass spectrometry on all proteins in conditioned medium of the aortic samples, a 10-fold increase of latent TGFβ binding protein 4 (LTBP4) in the BAV samples (Rocchiccioli et al., 2017). Histological analysis identified that, compared to normal aortic tissue, BAV dilated aortic tissue had increased levels of SMAD3 and TGFβ protein in the tunica media (Nataatmadja et al., 2013). However, when compared to dilated TAV aorta, the expression of SMAD 2/3 was higher in the TAV dilated aorta than the BAV dilated aorta (Rocchiccioli et al., 2017). Furthermore, it has been shown that the circulating TGFβ levels in BAV patient are elevated, which is in agreement with studies showing increased TGFβ signaling (Hillebrand et al., 2014; Rueda-Martínez et al., 2017).

Multiple studies have demonstrated that antagonizing TGFβ signaling in aneurysm mouse models prevents and even reverses aneurysm formation (Habashi et al., 2006; Ramnath et al., 2015; Chen et al., 2016). The positive effects of TGFβ antagonism on aneurysm formation were shown in using a neutralizing TGFβ-antibody or by blocking the AT1 receptor using Losartan, which also decreases TGFβ signaling. In different mice models, Fibrillin-1 deficient, Fibulin-4 deficient and ANGII treated mice, the TGFβ inhibition prevented and reversed aortic aneurysm, making it a promising target for therapy (Habashi et al., 2006; Ramnath et al., 2015; Chen et al., 2016). A study using cultured SMCs revealed that Losartan treatment decreased intracellular TGFβ protein levels and nuclear SMAD3 localization (Nataatmadja et al., 2013). BAV derived SMCs displayed a decrease in endoglin expression upon Losartan treatment (Lazar-Karsten et al., 2016). Furthermore, serum TGFβ levels decreased when mice were treated with Losartan. The same was also seen in Marfan patients treated with Losartan, validating the study results obtained in mice (Habashi et al., 2006; Matt et al., 2009). However, as mentioned above, so far Losartan treatment does not seem to decrease or prevent aneurysm formation in a clinical setting. Given the recent success of specific TGFβ blockers in other vascular disorders such as pulmonary arterial hypertension (PAH) and restenosis, targeting the TGFβ pathway more directly could be a strategy for developing new treatment modalities for TAA (Yao et al., 2009; Yung et al., 2016).

## Endothelial dysfunction in other diseases: implications for BAV-TAA?

Many cardiovascular disorders have highlighted the importance of normal endothelial functioning for maintaining homeostasis across the vessel wall, such as atherosclerosis, brain aneurysms, PAH, and hereditary haemorrhagic telangiectasia (HHT). PAH and HHT are 2 major genetic diseases in which the role of the endothelial cells is well recognized. Two recent advances in these research fields worth mentioning for future perspectives in BAV TAA research, will be discussed in the next paragraphs.

PAH is an incurable fatal disease caused by remodeling of the pulmonary arteries. Proliferation of the pulmonary artery smooth muscle cells (PASMCs) causes narrowing and occlusion of the lumen, leading to an increased pressure in the lungs and increased load of the right ventricle (Morrell et al., 2009). While originally defined as a SMC disorder, over the past years dysfunction of the endothelial cells has become of interest in the pathogenesis of PAH (Morrell et al., 2009; Sakao et al., 2009; Xu and Erzurum, 2011). The application of conditioned medium from normal endothelial cells to PASMCs resulted in an increase in PASMC proliferation rate (Eddahibi et al., 2006). This effect is exaggerated when adding conditioned medium of endothelial cells from PAH patients. Complementary, PASMCs from PAH patients showed an increased proliferation to both endothelial cell conditioned media, compared with control PASMCs. Two of the major players identified within the conditioned medium are miR-143 and miR-145. These miRs have been demonstrated to highly impact the SMC phenotypic switch, inducing a contractile phenotype (Boettger et al., 2009). Expression of these two miRs is regulated by TGFβ and they have been shown to be secreted in exosomes (Climent et al., 2015; Deng et al., 2015). Intriguingly, in PAH mouse models as well as patient lung tissue and cultured SMCs, miR-143-3p expression is increased. Furthermore, miR-143^−/−^ mice developed pulmonary hypertension, a phenotype that was rescued by restoring miR-143 levels (Deng et al., 2015).

Interestingly, signaling from endothelial cells to SMCs concerning miR-143 and miR-145 has also been investigated in atherosclerosis research (Hergenreider et al., 2012). Transduction of HUVECs with the shear-responsive transcription factor KLF2, or exposure of HUVECs to flow caused an increase in miR-143 and miR-145, indicating a flow responsiveness of the miR-143 and miR-145 expression (Hergenreider et al., 2012). Additionally, endothelial cells secrete miR-143 and miR-145 in microvesicles which alter gene expression in SMCs. Moreover, when treating *ApoE*^−/−^ mice with endothelial secreted vesicles containing, amongst others, miR-143 and miR-145, the mice developed less atherosclerosis (Hergenreider et al., 2012). SMCs of miR143 and miR-145 knockout mice displayed increased migration and proliferation. In addition, analyses of the mouse aortas showed ECM degradation in the miR-143 and miR-145 deficient mice. These results support the findings of a role for miR-143 and miR-145 in inducing a contractile SMC phenotype (Elia et al., 2009). Furthermore, in TAA miR-143 and miR-145 were found to be decreased compared to non-dilated samples (Elia et al., 2009). The impact these miRs have on SMC phenotype, the expression regulation by flow and their secretion by endothelial cells as well as the decrease in TAA, makes them relevant and interesting for BAV TAA research. The first study toward BAV and miR-143 and miR-145 was recently published, describing a local decrease of miR-143 and miR-145 in the inner curve of the BAV aorta compared to the outer curve. Moreover, they also found altered miR expression affecting mechanotransduction (Albinsson et al., 2017).

Intriguingly, mechanotransduction has also been of interest in HHT research. HHT is a vascular disease characterized by frequent severe bleedings due to fragile and tortuous blood vessels. Disturbed TGFβ signaling plays a major role in the development of these malformed blood vessels. Eighty percent of HHT patients have a mutation in *ENG* (HHT1) or *ALK1* (HHT2; McDonald et al., 2015). The endothelial cell-SMC communication is disrupted in HHT, and recruiting and differentiation of SMCs falters causing improperly formed vessels. Disturbed mechanotransduction in endothelial cells has been shown to impact BMP/SMAD1/5 signaling as well as vessel stabilization in HHT (Baeyens et al., 2016b). By subjecting endothelial cells to shear stress, SMAD1 was activated. Moreover, decreasing either ALK1 or endoglin both inhibited the SMAD1 activation in response to flow. Interestingly, when co-cultured with pericytes, both ALK1 and endoglin were found to be crucial for endothelial shear stress induced migration and proliferation of these pericytes (Baeyens et al., 2016b). It would be highly interesting to investigate if BAV endothelial cells also have an intrinsic mechanotransduction defect causing the aorta to be prone to TAA development. The study by Albinsson and colleagues showing the altered miR related to mechanotransduction in BAV aorta samples is an important first step to lead the BAV TAA research field toward relevant studies on mechanotransduction defects possibly explaining (part of the) BAV TAA pathogenesis.

## Conclusions and future perspectives

BAV is a common congenital cardiac malformation and the majority of BAV patients develop TAA over time. Although the last decade has witnessed the discovery of several key findings in the field of BAV-associated TAAs, the cellular and molecular mechanisms in BAV-associated TAAs that drive the degeneration of media of the vessel wall are still largely unknown. Many studies have focussed on changes in the signaling pathways in SMCs, however the importance of endothelial cells and their contribution to the initiation and progression of BAV-associated TAAs has not been appreciated in detail.

Under normal physiological conditions, endothelial cells and SMCs communicate with each other for optimal function of the vessel wall in order to maintain homeostasis in the circulatory system. Dysregulation of this communication can lead to medial degeneration and aortic aneurysm, clearly demonstrated in animal models using ANGII infusion or eNOS uncoupling. Interestingly, blocking TGFβ signaling is a possible treatment option to prevent TAA formation, as evidenced by multiple animal studies mentioned before. Patient samples also indicate a pivotal role for these pathways as revealed by the dysregulation of eNOS, Notch1 and TGFβ signaling proteins in the BAV aortic tissue. The involvement of these pathways is validated by the mutations that have been shown to cause BAV and/or TAA in mouse models and the finding of mutations in these genes in patients with BAV and TAA. In addition to these observations made *in vivo, in vitro* studies using patient derived endothelial cells indicate an EndoMT defect in cultured cells from BAV patients. In conclusion, all studies to date indicate great potential of an underexplored research field concerning the endothelial-smooth muscle cell communication in the BAV TAA formation.

While hardly studied in BAV, the importance of endothelial functioning for vessel homeostasis has been elucidated in other vascular disorders such as PAH, HHT, and atherosclerosis. In line with the latest research in these fields, it would be very interesting to investigate if the mechanotransduction and/or microvesicle secretion is altered in endothelial cells of BAV TAA patients. Unfortunately, research toward endothelial cell contribution in BAV TAA pathogenesis has been hampered by the difficulty of obtaining non-end stage study material. The discovery of circulating endothelial progenitor cells (EPCs) and endothelial colony forming cells (ECFCs) will provide a new study model, facilitating patient specific analysis of the endothelial contribution to the disease (Asahara et al., 1997; Ingram et al., 2004). Thus far, one study was published using these circulatory cells from BAV patients. An impaired EPC migration and colony formation potential was shown when the cells were isolated from BAV patients with a dysfunctional valve compared to BAV patients with a normal functioning valve (Vaturi et al., 2011). Currently, the cause and effect of impaired EPCs is unknown, and more research is required to understand the full potential of circulating EPCs and ECFCs in BAV TAA pathogenesis and their use as a biomarker for patient stratification.

**Figure 3:**
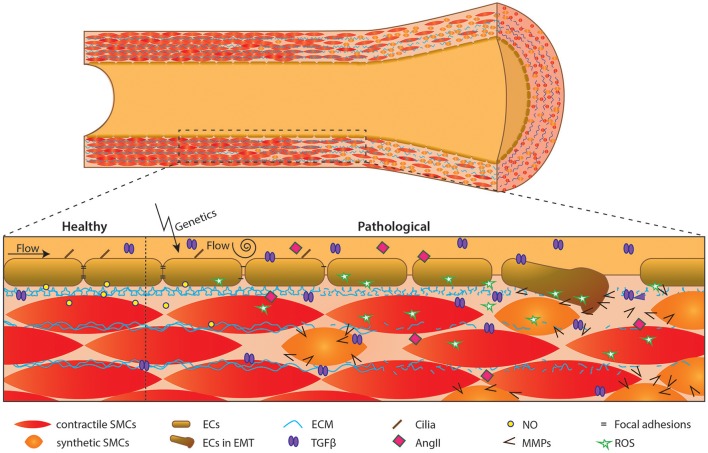
Schematic overview of events in development of aortic dilation. Schematic overview of an aorta over time. Initiation by flow and/or genetics causes endothelial cell dysfunction, affecting the aortic structure i.e., causing synthetic SMCs and lamellar fragmentation.

Although few studies on the role of endothelium in BAV disease and its associated TAAs have been performed in the last decade, some seminal papers have been published. In this review we have created an overview of the recent studies implicating endothelial cells as a pivotal player of vascular homeostasis, and their underappreciated role in TAA pathogenesis in patients with a BAV. Figure 3 schematically depicts the different factors and processes involved in BAV TAA development as discussed throughout this review. Up to date, we are still unable to stratify and cure BAV patients with TAA patients. Therefore, further research is required to understand the role of endothelial cells and comprehend the interplay between endothelial cells and SMCs in BAV-associated TAA. In conclusion, appreciation of the role of endothelium is crucial for a better understanding of BAV TAA pathogenesis, which is necessary in development of new therapeutic strategies for the BAV-associated TAAs.

## Author contributions

VvdP, KK, and M-JG conceptualized the review. All authors listed have made a substantial, direct and intellectual contribution to the work, and approved it for publication.

### Conflict of interest statement

The authors declare that the research was conducted in the absence of any commercial or financial relationships that could be construed as a potential conflict of interest.

## Footnotes

^1^Clinicaltrials.Gov (consulted 15-09-2017). *Identifier NCT01390181*.
